# Internet-based randomised controlled trials for the evaluation of complementary and alternative medicines: probiotics in spondyloarthropathy

**DOI:** 10.1186/1471-2474-9-4

**Published:** 2008-01-11

**Authors:** Sinead Brophy, Claire L Burrows, Caroline Brooks, Michael B Gravenor, Stefan Siebert, Stephen J Allen

**Affiliations:** 1 School of Medicine, Swansea University, Swansea, Wales SA2 8PP, UK

## Abstract

**Background:**

The clinical effectiveness of complementary and alternative medicines (CAMs) is widely debated because of a lack of clinical trials. The internet may provide an effective and economical approach for undertaking randomised controlled trials (RCTs) of low-risk interventions. We investigated whether the internet could be used to perform an internet-based RCT of a CAM fulfilling the revised CONSORT (Consolidated Standards of Reporting Trials) statement quality checklist for reporting of RCTs. A secondary aim was to examine the effect of probiotics compared to placebo in terms of well-being over 12 weeks.

**Methods:**

People aged ≥18 years with confirmed spondyloarthropathy living in the United Kingdom with internet access were invited to participate in an internet-based RCT of probiotic compared to placebo for improving well-being and bowel symptoms. The intervention was a probiotic containing 4 strains of live bacteria or identical placebo taken by mouth daily for 3 months. The primary outcome measure was the performance of the trial according to the revised CONSORT statement.

**Results:**

147 people were randomised into the trial. The internet-based trial of the CAM fulfilled the revised CONSORT statement such as efficient blinding, allocation concealment, intention to treat analysis and flow of participants through the trial. Recruitment of the required number of participants was completed in 19 months. Sixty-five percent (96/147) completed the entire 3 months of the trial. The trial was low cost and demonstrated that in an intention to treat analysis, probiotics did not improve well-being or bowel symptoms.

**Conclusion:**

The internet-based RCT proved to be a successful and economical method for examining this CAM intervention. Recruitment, adherence and completion rate were all similar to those reported with conventional RCTs but at a fraction of the cost. Internet-based RCTs can fulfil all the criteria of the revised CONSORT statement and are an appropriate method for studying low-risk interventions.

**Trial registration:**

ISRCTN36133252

## Background

The use of complementary and alternative medicines (CAMs) has increased considerably in the Western industrialised countries in the past decade. CAMs are often used for maintaining wellness and in addition conventional care for chronic and acute health conditions [1]. However, there is real debate around their use [2-4] because of the lack of high quality scientific evidence for their effectiveness. Randomised controlled trials (RCTs) are considered the highest form of scientific evidence for any primary research study. However, few RCTs have been performed on CAMs as there is little incentive for manufacturers to undertake the considerable financial investments required for phase 1–4 clinical trials that assess efficacy, effectiveness, safety and cost-effectiveness [5] when individual CAMs may be provided by several providers and can not be protected by patent. Also, many CAMs can be marketed without the legal restrictions placed on drugs. These barriers mean that the potential benefits of CAMs are generally not adequately assessed, and patients may be denied potentially effective and safe interventions from the national health service.

Internet-based trials may provide a highly economical way of overcoming many of these logistical and funding barriers. Internet-based trials can cut down on cost of staff time (including clinical costs and data entry time), allow frequent contact with participants, enable recruitment across a large area (national or international) instead of requiring multi-centred study design and less common conditions can be examined. We examined whether an internet-based RCT could generate high quality evidence regarding the effectiveness of a widely used CAM/food supplement in a specific patient population by evaluating the performance of the trial according to the revised CONSORT statement [6]

Our chosen intervention was probiotics in spondyloarthropathy (SpA a group of related disorders) as probiotics have an excellent safety profile [7] and are considered a food supplement. There is considerable evidence from clinical and epidemiological studies that inflammation of the gut is involved in triggering spondyloarthropathy [8-11] and in the severity of the resultant joint inflammation [12,13] Probiotics may help in treating this bowel inflammation [14-16] and are advertised for ankylosing spondylitis, the prototypic SpA on several commercial internet sites, despite the absence of any published clinical trials.

### Objectives

#### Primary

To examine if an internet-based RCT of a CAM can meet the revised CONSORT statement quality checklist for reporting of RCTs.

#### Secondary

To examine the effect of probiotic on well-being compared to placebo.

## Methods

### Participants

Participants were recruited from a link posted on the website of the national patient-led charity, the National Ankylosing Spondylitis Society (NASS). The trial web site provided information regarding the trial and stipulated that participants had to be 18 years or over, resident in the UK, have access to the internet and a diagnosis of SpA. Participants printed-out and signed a consent form and posted it to researchers. This included permission from the participant for the research team to contact their doctor (either general practitioner or rheumatologist) to confirm that they knew the participant, that the participant was more than 18 years old, had a diagnosis of SpA made by a rheumatologist and confirmed by X-ray or magnetic resonance scan, and did not have a immunosuppressive disorder such as HIV/AIDS or cancer. All the participants included in the study had a confirmation from their rheumatologist or GP that they had sacroiliitis as diagnosed using an X ray or MRI. Therefore, no participants have peripheral arthropathy only.

We also asked for the doctor to confirm other conditions such as iritis, psoriasis or inflammatory bowel disease. Participants were asked to stop taking live yogurts or other probiotic preparations for the duration of the study. Participants were encouraged to phone the research team if they required further information.

### Interventions

The probiotic (10 g lyophilized powder containing live bacteria: *Lactobacillus salivarius *(CUL61) 6.25 × 10^9 ^cfu (colony forming units), *Lactobacilllus paracasei *(CUL08) 1.25 × 10^9^cfu, *Bifidobacterium infantis *(CUL34) 1.25 × 10^9 ^cfuand *Bifidobacterium bifidum *(CUL20) 1.25 × 10^9 ^cfu) and the placebo (10 g maltodextrin) capsule were the same colour, size, smell and contained powder of identical appearance. Capsules were sent through the post and participants were instructed to keep the capsules in the refrigerator and take 1 capsule by mouth daily for 3 months.

#### Outcome measures

Outcome 1: to fulfil the revised CONSORT statement for reporting of parallel-group randomised trials for an internet-based RCT of a CAM (probiotics).

Outcome 2: Comparison of probiotic and placebo group in terms of well-being, bowel symptoms and arthritis severity (disease activity and function) after 12 weeks of treatment.

#### Sample size

We required a sample size of 140 participants for 95% power at 5% significance level (two sided) to detect a clinically relevant difference [17] in wellbeing of 1.5 (standard deviation 2.6) allowing for a 30% drop out rate. The analysis was by intention to treat, with participants being analysed according to the group to which they were randomised. No interim analyses were planned or performed.

### Random allocation

#### Sequence generation

A random allocation sequence, without the use of blocks, was generated by the manufacturer of the probiotic intervention (Cultech Ltd., Port Talbot, UK) using a computer generated random number sequence. The sequence allocated participants on a 1:1 basis to either the probiotic or placebo arms of the study.

### Allocation concealment

The allocation sequence was held by the manufacturer and was not accessible to the researchers until all data had been collected, analysed, interpreted and findings given to an independent researcher. The study team allocated participants consecutively to the randomization number sequence and, thereby, to the assigned trial intervention. The individuals generating the random number sequence were in a different site to those recruiting participants and posting out the intervention, and no members of the research team met any of the participants.

### Blinding

Participants and researchers were blinded to group assignment. No member of the research team had face-to-face contact with any of the participants.

### Statistical methods

The secondary end points were compared using general linear models, taking into account baseline values, age, sex and disease duration. Patients with complete and missing data (i.e. missing questionnaires) were compared to examine whether the assumption of data missing in a non-random way could be rejected. At the end of the study the manufacturers informed the research team which participants were in Group A or Group B but the identity of these groups was only revealed after analysis had been completed and the results submitted to an independent third-party. All patients who had completed at least the baseline questionnaire were analysed in an intention to treat analysis.

### Follow-up

Baseline data was collected using the secure trial internet site and included sex, age at onset of disease symptoms, flare of symptoms in the past 7 days and medication. On the same day each week for 3 months, participants completed a 10 cm visual analog scale for the self assessment of well-being [18], bowel symptoms (diarrhoea, stomach pain, blood in stools), disease activity (pain, discomfort, morning stiffness, fatigue and tenderness, all averaged to give a composite score [19]) and function [20,21]. There was also space to make additional comments regarding their health or the trial intervention. Participants reported on the number of study capsules that they had taken during the previous week. Each week patients were contacted by e-mail to remind them to complete the on-line questionnaire. If they did not complete a questionnaire they were contacted by e-mail to find out why.

### Ethical approval

Granted by London MREC ref 04/2/18 in 2004

## Results

This internet-based RCT of a CAM (probiotic) was able to fulfil the revised CONSORT statement quality checklist for reporting of RCTs (Table 1)

**Table 1:** How an internet-based trial can meet the quality criteria of the revised CONSORT statement

**PAPER SECTION**	**Description**
*TITLE AND ABSTRACT*	How participants were allocated to interventions is comparable to traditional RCTs
*INTRODUCTION *Background	Scientific background and explanation of rationale is comparable to traditional RCTs
*METHODS *Participants	Eligibility criteria for participants needs to be confirmed by a third person as the researchers do not see the participants. In 159 out of 160 consent forms a reply was obtained from the participants' primary care physician or rheumatologist. Settings and locations are generally online and remote access in an internet-based trial.
Interventions	Interventions in an internet-based trial can only be those that the participant administers themselves and are stable enough to be sent in the post. The probiotic intervention fulfilled both these requirements.
Objectives	Specifying objectives and hypotheses is comparable to traditional RCTs
Outcomes	Outcome measures for an internet-based RCT generally need to be self assessment measures. However, postage of samples (such as blood samples taken at the local hospital or primary care practice) could be feasible. The use of internet-based questionnaires to assess disease severity in SpA has previously been validated[21]
Sample size	Determination of sample size, stopping rules and interim analysis are comparable to traditional RCTs
Randomization – sequence generation	Randomization is comparable to traditional RCTs
Randomization – allocation concealment	Allocation concealment is easier with an internet-based RCT as the researchers never meet the participants and can only randomise after the details of the participants have been entered into the data collection system. Researchers did not know who was in group A or B until after all data had been collected and the database cleaned. The identity of group A or B was not revealed until after all the analysis was completed. Participants never knew if they were in group A or B.
Randomisation – implementation	The allocation sequence was generated by different individuals to those recruiting and to those giving the medication to participants. No member of the research team met the participants
Blinding (masking)	Blinding is feasible using internet-based trials as participants are unlikely to meet in order to compare treatments, researchers never meet participants so have limited ability see effects of treatments and the analyst can be kept completely blinded as the database does not contain any reference to allocation groups until all data collection and data cleaning has been completed.
Statistical methods	Statistical methods are comparable to traditional RCTs
*RESULTS *Participant flow	Participant flows are comparable to traditional RCTs. However, participants can drop out without giving reasons. Therefore perhaps additional measures to follow people such as telephone contact, is needed.
Recruitment	Dates defining the periods of recruitment and follow-up is comparable to traditional RCTs
Baseline data	The characteristics collected are all self reported but can be validated by a third person such as the participants medical practitioner
Numbers analyzed	Internet-based trial must use intention to treat as there is no way of assessing compliance. Internet-based trials can measure pragmatic effectiveness and not efficacy
Outcomes and estimation	Analysis presentation is comparable to traditional RCTs
Ancillary analysis	Analysis for an internet-based RCT is comparable to that in traditional RCTs
Adverse events	Adverse events are harder to report in an internet-based RCT than in a traditional RCT. There is reliance on the participants to report adverse events. This is the reason why internet-based trials can only be conducted on safe interventions.
*DISCUSSION *Interpretation	Interpretation of an internet-based RCT is comparable to that of a traditional RCT
Generalizability	Generalizability may be affected as participants are a very selected sample. Participants need to have access to the internet/e-mail, the knowledge to use the internet/e-mail and the motivation to self refer to join a trial. However, generalizability of traditional RCTs is compromised by the artificial environment of frequent clinical visits, this bias does not apply to the internet-based RCT.
Overall evidence	Overall evidence in an internet-based RCT is comparable to that in a traditional trial

### Participants

**Figure 1:**
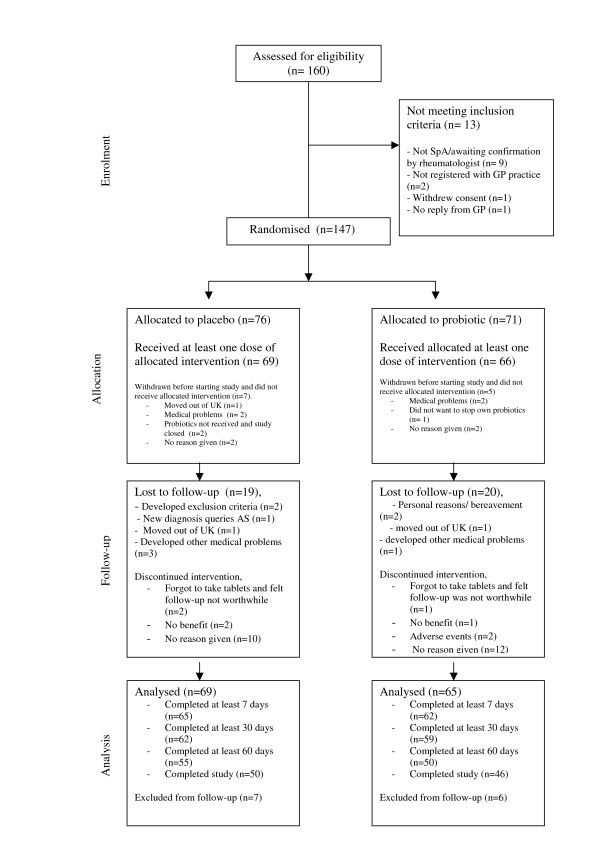
Flow diagram of the progress through the probiotics trial.

Of 160 consent forms received, 147 (91.9%) were eligible and were randomised (See Figure 1). Of 160 people giving consent, only 1 person was excluded because of no reply from their primary care practitioner or rheumatologist (See Figure 1). There were 11 who we could not get the diagnosis of SpA independently confirmed and one withdrew consent. Participants were recruited from throughout the UK including; Aberdeen, Brighton, Cornwall, Huddersfield, London and Cardiff and all had the opportunity to phone the researcher to discuss the study prior to consent. Participants showed good adherence to completing the weekly on-line questionnaire, with 65% (96/147) completing the study for the entire 3 months. If we remove the participants who were excluded (as a result of moving out of the UK, or developing other medical conditions) then adherence to completing the questionnaires was 70%.

### Recruitment

The website was made available to participants on 1^st ^September 2004 and the site was closed 19 months later (March 2006), when 147 participants had been randomised.

### Baseline data

The two groups were well balanced for baseline characteristics (see Table 2). However, the probiotic group appeared to have poorer function and global well-being, and used more non-steroidal anti-inflammatory drugs (NSAIDs).

**Table 2:** Baseline characteristics of participants^1^

	**Placebo (n = 69)**	**Probiotic (n = 65)**
Age (s.d)	42.7 (12.7)	44.8 (12.1)
Disease duration (s.d)	20.3 (13.4)	20.3 (13.2)
Male (%)	45 (65.2)	49 (75.4)
Iritis^2 ^(%)	13/58 (22.4)	14/52 (26.9)
Inflammatory bowel disease^2 ^(%)	6/58 (10.4)	2/52 (3.9)
Medication		
■ non-steroidal anti-inflammatory drug (%)	44/66 (66.7)	53/62 (85.5)
■ steroid (%)	2/67 (3.0)	0/63 (0.0)
■ disease modifying antirheumatic drug (%)	8/67 (11.9)	5/63 (7.9)
Global Well-being (scale 0–10)	3.2 (2.0)	4.1 (2.5)
Disease activity (scale 0–10)	3.5 (1.9)	4.1 (2.2)
Function (scale 0–10)	3.1 (2.5)	4.2 (3.0)

Notes

1. Values are mean (standard deviation) for continuous variables and number (percentage) for categorical variables

2. Confirmed by the participant's general practitioner or rheumatologist.

There was no significant difference in the baseline characteristics of participants who did not complete the entire 3 months trial and those who were lost to follow-up (Those completing the trial were 71.6% male (68/95), average age 43.9, disease duration 20 years compared non-completers who were 64% male (25/39), age 41 and disease duration 19 years). Therefore, the last recorded measurement was carried forward for analysis for all participants. There was no significant difference in the number of patients stopping/starting doses of concurrent AS medication in the placebo compared to the intervention arm.

#### CAM Outcome

There was no statistically or clinically significant difference between placebo and probiotic groups in terms of global well-being, bowel symptoms or severity of arthritis. The estimated probiotic effect was a worsening in well-being of 0.16 units on the 0–10 scale (with 0 being best and 10 being worst), with the 95% confidence interval (-0.6 to 0.93) well outside the bounds of clinical relevance (a required change in well-being of approximately 1.5 units) (See Table 3 for details and other outcome measures).

**Table 3:** Outcome variables

		**Placebo**			**Probiotic**		Estimated Probiotic Effect*
	Baseline	Final	% change	Baseline	Final	% change	Change in scale (95% CI). A positive value indicates a worsening in condition.
**Intention to treat **(n = 69 & n = 65)							
Global wellbeing (0–10 scale)	3.2 (2.0)	2.9 (2.3)	9.4%	4.1 (2.5)	3.7 (3.0)	9.8%	0.16 (-0.61 to 0.93)
***Bowel symptoms***							
Diarrhoea (0 – 10 scale)	1.9 (2.7)	1.0 (1.7)	47%	1.8 (2.6)	1.4 (2.3)	22%	0.24 (-0.36 to 0.83)
Stomach pain (0–10 scale)	2.1 (2.6)	1.2 (1.6)	42%	2.0 (2.7)	1.4 (2.2)	30%	0.17 (-0.42 to 0.76)
Blood in stools (0–10 scale)	0.6 (1.8)	0.5 (1.6)	17%	0.6 (1.6)	0.4 (1.1)	33%	-0.14 (-0.55 to 0.27)
***Arthritis severity***							
Disease activity (0–10 scale)	3.5 (1.9)	2.9 (2.2)	17%	4.1 (2.2)	3.6 (2.6)	12%	0.20 (-0.47 to 0.86)
Function (0–10 scale)	3.1 (2.4)	2.8 (2.6)	9.7%	4.2 (2.9)	4.0 (3.2)	5%	-0.04 (-0.50 to 0.43)

* General linear model, with probiotic effect adjusted for age, sex, disease duration and baseline levels

#### Adherence to medication

Of those who started the study (69 placebo and 66 probiotic), 3938 out of a possible 5796 (67.9% placebo) and 3748 out of a possible 5544 (67.6% probiotic) tablets were reported to have been taken.

#### Adverse events

11 people reported adverse events that they felt could be due to the trial interventions, 5 in the placebo group and 6 in the probiotic group. In the placebo group there were reports of stomach cramps (3), indigestion (1) and general decline in well being (1). In the probiotic group there were reports of stomach cramps (3), indigestion (1), painful spots (1) and dizzy spells (1). In two cases the participants stopped the trial medication and reported by e-mail that the symptoms had continued.

## Discussion

This internet-based trial of a CAM worked well as assessed by the revised CONSORT statement (Table 1) [6]. The required number of participants were recruited from a wide geographical area in a reasonable time frame and 92% of respondents were eligible for the trial. General practitioners and rheumatologists confirmed participant's identity and assessed eligibility for the trial without payment, suggesting a high level of support for this approach to clinical research among clinicians.

Participants' compliance with the trial procedure was satisfactory with 65% submitting all the required on-line questionnaires and a reported 68% compliance with the trial intervention. Future internet-based RCTs could include more objective assessments than self reporting. For example, participants could submit biological samples by post for detection of the intervention.

Participation in internet-based trials requires internet access and self motivation to access the research website, participants need to read the study information and then submit a consent form. This might limit generlisabiltiy as they may not be representative of the wider community. However, the fact that the participants have themselves initiated their involvement in the study demonstrates motivation and may also encourage their compliance and thus, increase validity of the findings.

Other factors are likely to discourage compliance in internet-based clinical trials. Participants in traditional RCTs may perceive a benefit from face-to-face contact with health professionals and other members of the research team. Although this may encourage compliance, it is labour intensive and creates a somewhat artificial environment for testing an intervention and the internet approach may better stimulate the "real world" regarding compliance with interventions. Thus, traditional RCTs may be more suitable for explanatory RCTs and the internet-based approach may be suitable for pragmatic RCTs. Methods to increase compliance in internet studies might include telephone contact with researchers, a requirement to post back unused capsules, and alarms or reminders that the intervention is due. Compliance in the probiotic group may have been considerably higher if it had been effective in reducing symptoms and improving well being. Thus, generalizability of traditional RCTs suffers from the artificial trial environment which does not occur in internet-based RCTs [22].

The lack of direct contact with participants compromises follow-up for adverse events. All of the published internet-based studies to date have tested low-risk interventions. They have included a trial of kava and valerian for anxiety and insomnia [23], topical ointment for herpes labialis [24] and a glucosamine for knee pain in osteoarthritis [25]. Probiotics have an excellent safety record [7]. Therefore, we considered this preparation to be suitable for testing using this approach.

This trial was funded by a grant for £4977 from NASS which covered the cost of the data collection, researcher time, postage of medication and letters to doctors and analysis (probiotics were provide free of charge and writing up time was not included in funding). This compares favourably with the costs of traditional RCTs.

In this study the probiotic preparation was not found to improve well-being, disease activity or function compared to placebo. However, it could be argued that the sample tested had long term advanced disease and probiotics may be may be beneficial in the early stages of SpA or for people with milder disease. In addition, it is possible that a larger dose or different mixture of strains of probiotic may have had a beneficial outcome.

In conclusion, we consider that internet-based RCT's are an effective and economical way to test low risk interventions where close follow-up for adverse events is not required. This approach may be particularly suited to CAMs, where traditional RCT's are unlikely to be undertaken, to deliver the evidence-base required by the regulatory authorities [3,4,26]

## Conclusion

An internet-based approach allowed us to perform a high quality RCT according to the revised CONSORT statement at low cost. Internet-based trials may be a cost-effective approach for assessing low-risk interventions. In this study probiotics showed no statistical or clinical significant improvement in SpA.

## List of abbreviations

CAMs : complementary and alternative medicines

CONSORT: Consolidated Standards of Reporting Trials

NASS: National Ankylosing Spondylitis Society

NSAIDs: non-steroidal anti-inflammatory drugs

RCT: Randomised controlled trial.

SpA: spondyloarthropathy

## Competing interests

The author(s) declare that they have no competing interests.

## Authors' contributions

SB, SA and MG wrote and designed the protocol. CBu and SB collect the data with input and advice from SS and SA. CBo, SB and MG analysed the data. All authors were involved in interpreting and writing the final results and manuscript.

## Pre-publication history

The pre-publication history for this paper can be accessed here:


